# Genomic Dynamics and Functional Insights under Salt Stress in *Gossypium hirsutum* L.

**DOI:** 10.3390/genes14051103

**Published:** 2023-05-18

**Authors:** Zunaira Anwar, Aqsa Ijaz, Allah Ditta, Baohua Wang, Fang Liu, Sana Muhy-Ud-Din Khan, Sajjad Haidar, Hafiz Mumtaz Hassan, Muhammad Kashif Riaz Khan

**Affiliations:** 1 Nuclear Institute for Agriculture and Biology College (NIAB-C), Pakistan Institute of Engineering and Applied Sciences (PIEAS), Islamabad 45650, Pakistan; zunaira.anwar738@gmail.com (Z.A.); aqsaijaz1995@gmail.com (A.I.); adbotanist@yahoo.com (A.D.); smkhan6464@gmail.com (S.M.-U.-D.K.); sajjadhaidar_pk@yahoo.com (S.H.); hassan_niab@yahoo.com (H.M.H.); 2 Nuclear Institute for Agriculture and Biology (NIAB), Faisalabad 38000, Pakistan; 3 School of Life Sciences, Nantong University, Nantong 226000, China; 4 State Key Laboratory of Cotton Biology, Institute of Cotton Research, Chinese Academy of Agricultural Science, Anyang 455000, China; liufcri@163.com

**Keywords:** salt tolerance, cotton, marker-assisted selection, genotyping by sequencing, genome wide association, single nucleotide polymorphism

## Abstract

The changing climate is intensifying salt stress globally. Salt stress is a menace to cotton crop quality and yield. The seedling, germination, and emergence phases are more prone to the effects of salt stress than other stages. Higher levels of salt can lead to delayed flowering, a reduced number of fruiting positions, shedding of fruits, decreased boll weight, and yellowing of fiber, all of which have an adverse effect on the yield and quality of the seed cotton. However, sensitivity toward salt stress is dependent on the salt type, cotton growth phase, and genotype. As the threat of salt stress continues to grow, it is crucial to gain a comprehensive understanding of the mechanisms underlying salt tolerance in plants and to identify potential avenues for enhancing the salt tolerance of cotton. The emergence of marker-assisted selection, in conjunction with next-generation sequencing technologies, has streamlined cotton breeding efforts. This review begins by providing an overview of the causes of salt stress in cotton, as well as the underlying theory of salt tolerance. Subsequently, it summarizes the breeding methods that utilize marker-assisted selection, genomic selection, and techniques for identifying elite salt-tolerant markers in wild species or mutated materials. Finally, novel cotton breeding possibilities based on the approaches stated above are presented and debated.

## 1. Introduction

Plant biofibers are extremely valuable in terms of economics and trade. The most important fiber-producing crops are cotton (*Gossypium hirsutum* L.), jute (*Corchorus capsularis* L.), kenaf (*Hibiscus cannabinus* L.), flax (*Linum usitatissimum* L.), and hemp (*Cannabis sativa* L.) [1,2]. The fibers produced from these plants are of excellent quality and have a high economic value globally [3,4]. Cotton, the most significant crop, serves as the foundation of the textile sector [5,6]. Both cotton fiber and seed have commercial applications [7]. Cotton byproducts are used for a variety of purposes, including as oil, feed, food products, biofuels, and textile materials [8,9]. 

Climate change has resulted in increased soil salinity [10]. The rise in sea level due to climate change has increased salinity in soil by up to 33 percent from the last 25 years [11,12,13]. Global warming is increasing, resulting in glaciers and ice sheets melting, and the thermal expansion of sea water leads to a rise in sea levels. The obvious outcomes of a rising level are flooding and increased salinity. The latter is seen in increasing salinity in ground as well as surface water via mixing saltwater with fresh water. Salinity is more common in arid regions than in semi-arid regions [14,15]. Heavy/low rainfall and repeated drought conditions have been common because of shifts in weather. Both excessive and insufficient rainfall can influence soil salinity. Abundant rainfall can lower the concentration of salts in the soil by diluting them, resulting in decreased salinity. This is because the rainwater rinses away some of the salt in the soil, creating a less concentrated solution. Conversely, insufficient rainfall can cause an increase in salinity. This is because the salt in the soil is not washed away, resulting in the accumulation of salt over time. Typically, soil salinity is more likely to rise in arid regions with limited rainfall or in locations where the water table is high, enabling saltwater to seep into the soil. The water moves upward and increases salt in the root zone areas of coastal and shallow water table regions. Moreover, soil salinity is affected by periodic episodes of temperature as well as rainfall [16]. 

Salt stress is the second most prevalent abiotic stress after drought, impairing plant growth and reducing agricultural production worldwide [17,18]. Plant salt stress is a phenomenon that occurs when soil solution contains an excessive amount of salts, leading to the inhibition of plant growth or even death. On a global level, excess salt is the most significant factor that inhibits plant growth. This condition can hinder the absorption of vital nutrients and water required for plant growth, resulting in stunted growth and lower yields [19,20,21]. It can also inflict harm on plant roots, leaves, and other organs, decrease photosynthesis, and disturb the plant’s metabolism [22,23,24].

The world is facing a concerning issue of declining arable land, which has led to heightened competition for grain and fiber crops [7,25]. However, the presence of salts in these soils poses challenges to cotton growth and development, as it disrupts crucial physiological and biochemical processes [26,27]. Moreover, the early developmental stages of cotton are particularly vulnerable to salt stress, which has a significant impact on eventual crop output [28].

Due to poor management practices and lack of regulation, salt stress is getting worse every year. Saline irrigation increases the amount of sodium chloride in the soil, which can lead to soil degradation [28]. Cotton plants are affected in multiple ways by salt stress, including diminished growth, limited leaf area expansion, and impaired nutrient uptake. The accumulation of cytoplasmic Na^+^ and Cl^-^ ions, which can lead to cell death, is also a consequence of salt stress [13]. Furthermore, it can decrease the activity of metabolic enzymes, contributing to the deterioration of fiber quality [29].

Economically, cotton is a substantial fiber crop that accounts for a large share of the any country’s GDP [30]. Addressing the salinization problem remains a challenge; therefore [31], conventional breeding interventions in cotton have been successful in improving salt tolerance and have doubled the productivity of cotton. This is achieved by exploiting the global gene pool, producing novel variations through hybridization, and selecting and stabilizing new varieties for local adaptation. However, conventional breeding technologies require laborious selection processes, which are time-consuming and limited in their effectiveness [32]. In addition, advanced techniques, including marker-assisted selection and genomic selection, are now available to facilitate the selection of salt-tolerant varieties more efficiently. Additionally, various biotechnological techniques can be used to induce mutations and introduce novel traits into the cotton genome, thereby enabling the development of cotton varieties that are more resilient to salt stress [33].

## 2. Impacts and Responses of Salt Stress on Cotton Plant

A potential avenue for improving cotton performance in saline environments could involve gaining an understanding of how cotton responds to salt, its resistance mechanisms, and effective management approaches. This knowledge could inform the development of strategies to enhance cotton growth and yield in such environments [34].

Salt stress decreases biomass production, stem thickness, reduction in leaf area, root and shoot weight, and yield of seed [35]. Cotton yield decreases at a salinity level of 7.7 dS m^−1^, and a 50% reduction in output was noted at 17.0 dS m^−1^ [36]. Under salt stress, fiber strength, length, and micronaire values decrease in both *Gossypium hirsutum* and *Gossypium barbadense*, but ginning out-turn increases. However, this increase is accompanied by a decrease in fiber strength, length, and micronaire values in both *Gossypium hirsutum* and *Gossypium barbadense* [35]. In addition, salt stress also decreases the photosynthetic activity and percentage of carotenoid contents, ultimately resulting in poor plant growth. Compared to later stages, salt stress is more detrimental to the germination, emergence, and seedling phases [30]. Salt stress can lead to delayed flowering, a decrease in the number of flowers per plant, an increase in fruit shedding, and a reduction in boll weight. Under salt stress, the concentration of Na^+^ and Cl^+^ increases by decreasing the K^+^, Ca^2+^, and Mg^2+^ concentration in cotton leaves. Increasing certain ions can decrease other ions due to competition for uptake by the plant. When a plant is exposed to high concentrations of some ions, such as sodium and chloride ions, they compete with other ions, such as potassium and magnesium, for uptake by the plant. This can result in decreased uptake of the other ions, leading to a decrease in their concentrations in the plant. This competition between ions for uptake is known as ionic competition and can have a significant effect on the overall ion concentrations in the plant [37].

Na^+^ exclusion has commonly been attributed to salt tolerance in cotton. Cotton is affected by high salinity, resulting in reduced uptake of potassium (K) and nitrogen (N), whereas low salt levels have minimal impact on their absorption [35]. Reduction of metabolic enzyme activity, such as alkaline invertase, sucrose phosphate synthase, and acidic invertase results in low fiber quality under salt stress. For example, Peng and others in 2016 discovered that in two different cotton cultivars, high soil salinity hindered cellulose synthesis, decreased the rate of sucrose conversion, and affected the functions of sucrose-metabolizing enzymes [38].

### 2.1. Impact of Salt Stress on Cotton Growth

To address the salt stress issue, it is important to comprehend how salt affects cotton at various growth stages. 

#### 2.1.1. Root and Shoot

Salt stress is more common in cotton at germination, emergence, and young seedling stages [35]. However, salt stress is more sensitive in seedlings at germination stage than in seedlings at the juvenile stage [34]. A significant reduction in cotton production occurs when there is a decrease in the plant population due to poor germination [39]. The growth of roots is impeded by salt stress as it decreases the number of secondary roots and diminishes the length of roots [40]. Primary root length is reduced with high salt concentration, while secondary root length is similarly slowed by modest salt stress [41]. Root growth is variably reduced according to soil type as salt stress increases. The effects are more obvious in clay and loam soils than in sandy soils [42]. High salt stress has a detrimental impact on vegetative development. Salt stress lowers the ratio of shoots to roots, indicating that shoot growth is more susceptible to salt stress as compared to root growth [34]. Studies conducted at different stages of cotton growth have found that the six-leaf stage is particularly susceptible to the negative impacts of salt stress [43].

#### 2.1.2. Boll Development and Yield

As salt stress increases, cotton yields decrease drastically, which is evidenced by a decrease in the number of bolls and their weight. Furthermore, a reduction in the number of fruit-bearing positions, a delay in blooming, an increase in flowers shedding, and a decrease in the number of bolls per plant due to salt stress all contribute to a reduction in mature bolls [34]. Detrimental impacts of high salt stress on vegetative development eventually delay flowering and might also cause a delay in flower blooming. Irrigating cotton with highly saline water during the budding stage can result in a yield reduction of approximately 90 percent [34].

#### 2.1.3. Fiber Quality 

Fiber quality traits are genetically controlled but are influenced by the environment [44,45]. Fiber length, strength, and maturity are all reduced under salt stress, whereas fiber fineness increases. It has been reported that when the Na^+^ ion percentage is increased, it negatively affects the fiber length, strength, and micronaire values [37,46]. In salt-sensitive cultivars, cellulose content and sucrose transformation rate both dropped considerably with an increase in NaCl level, resulting in fiber quality degradation. Sucrose is accessible in a saline environment, but due to reduced activity of metabolic enzymes such as sucrose phosphate synthase, acidic invertase, and alkaline invertase, it is not effectively transformed into cellulose [38]. Table 1 represents the findings of salt stress effects during various growth stages in cotton.

### 2.2. Response of Cotton Plant to Salt Stress

Under conditions of salt stress, soluble salts accumulate in the root zone of cotton, leading to the development of osmotic and ionic stress, as well as disturbances in mineral balance [68], which result in a severe decrease in crop quality and production [69]. Because of osmotic, ionic, and oxidative stressors, salt stress severely affects cotton growth, development and production. As a result, identifying and developing cotton cultivars that can withstand salt stress is a major challenge for sustainable agriculture [70].

Cotton’s most effective response to salt stress either excludes excess sodium or compartmentalization. There is significant potential to create salt-tolerant cotton cultivars by boosting enzymatic and nonenzymatic antioxidant gene expression. Additionally, priming seeds is an efficient method for enhancing cotton germination in saline soils [34].

Seed priming is an economical method of hydrating seeds and promoting rapid, uniform germination. This technique results in reduced imbibition time, increased metabolic activity, and osmotic adjustment. It also triggers molecular changes such as DNA synthesis, protein production, and the accumulation of antioxidants. There are various types of priming methods, including hydropriming (presoaking in water with or without drying), osmopriming (soaking in osmotic solutions such as sugar or mannitol followed by air drying), and hormopriming (soaking in hormone solutions such as auxin or gibberellic acid). These methods have been reviewed in multiple studies [71]. 

Zhang and fellows in 2021 divulged that melatonin priming can enhance the salt tolerance of *Gossypium hirsutum* L. (cotton) seedlings under salt stress conditions. According to the study, seedlings that were cultivated from seeds primed with 25 mM melatonin displayed greater root and shoot biomass and increased ion accumulation in comparison to the control group. These results suggest that melatonin priming has a beneficial effect on salt stress tolerance. The study also concluded that melatonin-primed seedlings performed better under saline conditions compared to nonprimed seedlings, indicating the potential for melatonin priming to enhance salt tolerance in cotton plants [69].

Shaheen and colleagues (2015) found that seed priming with KNO_3_ (1.5%) was found to reduce salt stress in cotton seedlings, improving dry matter and nutrient uptake, as well as shoot and root lengths, biomass, and cation (Ca^2+^, Na^+^, and K^+^) accumulation [72]. Wang and others (2021) also demonstrated that Mepiquat chloride-priming positively improve cotton seed germination and seedling establishment when exposed to salt stresses [73]. According to the report by Ahmadvand and fellows (2012), the priming of cotton seeds with KNO_3_ resulted in improved germination and seedling growth even when subjected to salt stress [74].

Utilizing marker-assisted selection (MAS) and exploiting the inter- and intravariation in cotton germplasm can be effective in generating salt-resistant variants. Additionally, a transgenic approach could serve as a crucial tool for cultivating cotton in saline conditions. Transgenic approaches involve transferring specific genes from one organism to another in order to achieve desired characteristics. Transgenic methods are quicker than traditional breeding techniques and can enable crossing of genera boundaries. Through the transfer of salt-responsive genes from other sources, transgenic approaches have been utilized to create salt-resistant plants. This technology has already demonstrated successful implementation in cotton [34].

Research studies have shown that the introduction of *TsVP*, a gene for H^+^-PPase from *Thellungiella halophilla*, into transgenic cotton plants can enhance their root and shoot growth, as well as their photosynthetic activity under high salt stress conditions [75]. This is likely the result of *TsVP* aiding the storage of Na^+^ and Cl^−^ in the vacuoles, which leads to a decrease in membrane ion leakage and malondialdehyde levels [75]. Expressing the *TsVP* gene from *Thellungiella halophila* can enhance cotton emergence, survival, and fiber quality under high saline conditions, while expression of the *AVP1* gene from *Arabidopsis thaliana* improves growth and fiber yield in salt-stressed transgenic cotton. Co expression of *AtNHX1* and *TsVP* genes in cotton also boosts emergence rate and yield under high saline environments [76]. In the future, researchers may utilize a combination of conventional techniques and state-of-the-art molecular technologies to breed salt-tolerant plant varieties [34].

There is substantial inter- and intraspecific variation in cotton salt tolerance, which is critical for selection and breeding regarding salt stress [43]. In the context of saline stress, the process of ion exclusion, specifically the exclusion of Na^+^/Clˉ, is accountable for the uptake and storage of detrimental ions within the tissues of cotton [68]. Several studies have shown that increased levels of K^+^/Na^+^ and Ca^2+^/Na^+^ in cotton tissues are associated with greater tolerance to saline stress. For instance, Kumar and colleagues in 2020 observed varying levels of inorganic sodium (Na^+^) accumulation in different cotton genotypes. The salt tolerant genotypes displayed higher potassium (K^+^)/sodium (Na^+^) ratios than their salt-sensitive counterparts [70]. Zafar and others during 2020 and 2021 discovered that tolerant cotton genotypes were able to maintain a stable potassium-to-sodium ratio in comparison to salt-sensitive cotton genotypes [27]. In 2003, Ahmad and colleagues conducted a study to investigate the effect of the calcium-to-sodium ratio for salt tolerance in plants. They reported that salt tolerant genotypes exhibited higher calcium-to-sodium ratios in their leaves than salt sensitive ones under saline conditions. The outcomes of the study indicate that calcium may have a pivotal function in the maintenance of proper membrane function and the regulation of its permeability, leading to normal growth in salt tolerant varieties in contrast to salt sensitive ones [77].

Consequently, this parameter can serve as a selection criterion for screening salt tolerant varieties. Genotypes demonstrating elevated antioxidant activity under saline conditions can be considered more tolerant to salt stress [78]. Genetic analysis of growth, fiber characteristics and yield under salt stress have shown to be genetically regulated via different quantitative trait loci (QTLs). Larger genetic additive variance of these traits can be utilized in cotton breeding programs for salt tolerance [79].

## 3. Classical Breeding in Cotton

Traditional breeding is used to develop new crop varieties/lines with desired traits via crossing closely or distantly related individuals [80]. Classical breeding exploits existing genetic diversity, primarily by way of homologous chromosomal recombination [81,82]. In order to produce plant varieties or hybrid plants that may not occur naturally, traditional plant breeders may employ in vitro techniques such as protoplast fusion, embryo rescue, or mutagenesis. Classical breeding of self-pollinating crops typically involves a variety of techniques, including introduction of new germplasm, selection using mass selection or pure line selection, hybridization followed by pedigree or backcross selection methods, and a single-descent technique. For cross-pollinated crops, the techniques used may include mass selection, development of hybrid varieties, and the creation of synthetic varieties [83,84].

According to Fita and coworkers in 2015, classical breeding for abiotic stress tolerance is considerably more difficult than breeding for other characteristics [85]. One reason is that the determination of characteristics is laborious and tedious which are highly associated with salt tolerance. Plants respond to various abiotic stresses differently and exhibit different levels of tolerance according to their phenological conditions [86]. Conventional breeding involves multiple stages of screening over multiple generations to identify the most suitable parents for crossing to develop consistent and high-performing varieties. This is achieved through continuous selection in various environmental conditions, using contrasting and target production environments to identify the genotypes with the best adaptation to specific or diverse conditions [87].

Conventional plant breeding involves several methods to identify stress-tolerant plants, such as field screening, phenotypic screening, biochemical and molecular markers, physiological screening, and hydroponic screening. Field screening involves growing different varieties of plants in areas that are likely to face stress and observing how they respond [88]. Phenotypic screening is carried out by observing visible plant characteristics, such as growth rate, flower color, or disease resistance, that may be associated with stress tolerance. Biochemical and molecular markers can help identify genes that code for enzymes involved in stress tolerance [89]. Under stress conditions, physiological screening involves assessing parameters such as photosynthetic rate, transpiration rate, and water use efficiency in plants. Hydroponic screening involves growing plants in a nutrient solution and subjecting them to different stress conditions, such as water scarcity or high salt concentrations. While these screening methods can be helpful in identifying tolerant genes and selecting better plants for further breeding, it is important to note that a plant may not show the same tolerance in the field that it would in the laboratory due to other factors such as pests or disease [90,91].

On the contrary, neglected crops and landraces have a diverse range of genetic variation as well as survival approaches along with a wide range of stress responses [92]. Farmers have chosen them for ages since they are tailored to a certain environment. As a result, landraces that originated under various environments have distinct adaptations to those conditions.

Genetic variance components, environmental interactions, heritability, and correlations between fiber characteristics have all been partially discovered using classical quantitative genetics research methodologies. Conventional breeding based on phenotypic selection has enhanced fiber quality along with fiber yield throughout the twentieth and twenty-first centuries [93]. In contrast, intensive selection for selected traits have caused a decline in the genetic diversity of commercially grown cotton. Classical plant breeding efforts have struggled to transmit novel, stable allelic diversity resulting from interspecific hybridization [93]. 

Most of the currently employed methods in conventional cotton breeding projects are the same as those used in the previous half-century, while the breeding technologies evolved substantially with the passage of time. So by coupling these conventional methods with advanced gene editing techniques such as insertion of desired genes using CRISPR can help to dig out the dilemma of declining cotton production [94,95].

In conventional breeding operations, an increase in lint production is still the primary selection criterion. To improve yield components, growth pattern, yield/yield stability, maturity, and plant resistance characteristics are frequently selected and evaluated [96]. New fiber testing technologies have enabled breeders to focus more on increasing fiber quality and making headway towards overcoming the formerly unfavorable correlations amid yield and fiber quality. Advancements in conventional cotton breeding programs have led to the identification and utilization of resistant germplasms. These resistant germplasms can help breeders to effectively harness and utilize the genetic diversity and identify genes responsible for desirable traits, which in turn can be used to develop improved varieties [33].

## 4. Screening Techniques for Evaluation of Salt Stress in Cotton Germplasm

To commence breeding programs for a crop species or its related species, exploration of the genetic diversity within the species is necessary. By screening genetically diverse germplasm, tolerant genotypes can be identified and utilized in a breeding program to produce crops with desired traits. This process can facilitate the development of new plant varieties with enhanced tolerance to various stresses [97]. Despite providing a more realistic outcome, screening for salt stress in soil is a challenging task, as it is complicated by spatial and temporal variability [98]. The presence of heterogeneous soil components and biotic or abiotic environmental factors can influence the effects of salinity. Therefore, laboratory-based salt screening tests are preferred over field screening, as they allow for effective control and monitoring of external factors such as humidity and temperature. Large-scale soil-based tunnel experiments or hydroponic experiments are recommended for assessing the effects of salinity. These methods provide a controlled environment that can be adjusted to similar conditions and are, therefore, suitable for evaluating salt tolerance in plants [99].

In the field of plant breeding, screening of germplasm is a critical step towards identifying and selecting genotypes with desirable traits, including salt tolerance. The use of solution culture in hydroponic or supported hydroponic systems has been the primary method for screening germplasm, with various systems such as gravel culture, sand culture, and soil-based systems grown in a greenhouse setting. The selection of an efficient screening system and growth culture methods, along with effective selection criteria, is crucial to ensure cost and time efficiency and the ability to screen large numbers of genotypes or accessions of a species with minimal labor and resources [98].

Efforts to develop salt tolerant cotton varieties have been ongoing for years, following the model for other crops. In 1974, Abul-Naas and Omran conducted one of the earliest screenings and found that *G. barbadense* was more salt tolerant than *G. hirsutum*. Akhtarand his fellows compared two screening methods in 2010, using 12 cotton genotypes, and found that the solution culture method was equally effective as the plant yield-based soil method for selecting and transitioning salt-tolerant genotypes to field testing [100].

In 2020, Sikder and his colleagues conducted a hydroponic experiment to investigate the salt tolerance traits in cotton genotypes during the seedling growth stage. The study demonstrated significant effects of salt stress on the evaluated traits, indicating considerable variation among the genotypes. The screening process categorized the genotypes into three groups, namely salt tolerant, moderately salt tolerant, and salt sensitive. Z9807, Z0228, and Z7526 were identified as the most salttolerant cotton genotypes, respectively, based on the screening results [70]. Castillo observed positive responses to salt stress in TX 307 and TX 310 cotton varieties using hydroponic technique. Bhandari screened 150 CRS accessions in both hydroponic and pot-based methods and found four lines that performed well in both systems. Bibi screened eight cotton genotypes at five NaCl concentrations and found significant differences among genotypes for various growth parameters [100].

In 2020, a hydroponic study was conducted by Sikder and his colleagues to assess salt tolerance traits in cotton genotypes at the seedling growth stage. The results indicated significant impacts of salt stress on the evaluated traits, showing considerable variation among the genotypes. The screening process classified the genotypes into three groups, consisting of salt tolerant, moderately salt tolerant, and salt sensitive. The screening results revealed Z9807, Z0228, and Z7526 as the most salt tolerant cotton genotypes, respectively [101].

In summary, the solution culture screening approach is equally effective as the soil-based methods in identifying and characterizing salt tolerant cotton genotypes. It is recommended that the initial selection of genotypes through solution culture experiments under controlled conditions, utilizing established physiological traits and criteria, can be a crucial step in the process of breeding and selecting salt tolerant cotton varieties [102].

## 5. Morpho-Physio and Biochemical Markers for Evaluation of Salt Stress in Cotton

Salt stress negatively impacts crop growth and development through water shortage, ion toxicity, and nutrient imbalance. Developing salt tolerant crops requires genetic variability and specific selection criteria. However, screening for salt tolerance can be challenging and time-consuming, requiring evaluation of large numbers of field crop accessions under both laboratory and field conditions [103]. 

Various crops have been screened for salt tolerance using a range of morphological, physiological, and biochemical indicators. Parameters related to both roots and shoots have been considered to evaluate variations in response to salt stress [104]. In 2019, Sharif and fellows found that high salt stress led to a decrease in both root and shoot length in cotton genotypes [34]. In 2010, Basal and in 2017, Yadav and Vamadevaia investigated the salt stress tolerance of cotton plants during the seedling stage and reported that tolerant genotypes displayed greater root and shoot lengths than susceptible genotypes. Salt tolerant genotypes exhibited less reduction in root and shoot length as compared to salt sensitive accessions, with shoots displaying higher sensitivity than roots [105,106]. Munwar and others in 2021conducted a study to evaluate salt tolerance in cotton germplasm during the early seedling stage. The results indicated that tolerant genotypes had greater fresh weight as compared to those susceptible [5]. Therefore, parameters related to root and shoot growth can be considered as crucial indicators for selecting against salt stress. This supports the results of a previous study [104]. 

Physiological markers are critical in identifying salt tolerance in plants, including the K^+^/Na^+^ ratio, which has been used as a reliable criterion for selecting salt tolerant cotton genotypes. Optimum K^+^/Na^+^ ion ratio is crucial for plant performance under salt stress. Chlorophyll degradation under salt stress reduces photosynthesis and plant growth, and high chlorophyll concentration is positively correlated with photosynthesis rate, dry matter production, and yield [107,108]. 

In response to salt stress, plants undergo rapid changes in osmotic parameters, including turgor pressure, osmotic pressure, relative water content (RWC), and water potential, which can be measured to evaluate the extent of stress. Cotton, for instance, exhibits reduced leaf relative water content when subjected to 200 mM NaCl. The effects of salt stress on osmotic changes can be measured by determining the levels of osmolytes, such as sucrose, proline, and glycine betaine, which accumulate in plants as a stress response. Salt-tolerant cotton genotypes typically exhibit higher levels of proline and glycine betaine content compared to salt-susceptible genotypes [109].

In the search for salt-tolerant genotypes, screening with biochemical markers has become crucial. Malondialdehyde (MDA) accumulation has been established as a reliable indicator of oxidative stress and membrane integrity and can distinguish between salt-tolerant and salt-sensitive genotypes, as demonstrated in previous studies by Demiral and Türkan during 2005 [110,111].

In a 2015 study, Guo and fellows observed increased lipid peroxidation in the root tissue of two cotton genotypes under salt stress. After relief from salt stress, the MDA content in “Lumianyan 37” roots decreased by 28.9–29.4%, compared to a decrease of 13.3–17.2% in “Sumian 22”. These results suggest that “Lumianyan 37” may have better protection against oxidative damage than “Sumian 22” under salt stress conditions [112]. In 2017, Wang and colleagues reported that salt-sensitive genotypes exhibited a higher MDA ratio than their salt-tolerant counterparts [113].

Salt stress disrupts electron transport and causes an oxidative burst, increasing reactive oxygen species (ROS) levels that can damage cells. ROS levels are regulated by the enzyme activity of ROS producers and scavengers. Under salt stress, various enzymes including peroxidase (POD), catalase (CAT), superoxide dismutase (SOD), monodehydroascorbate reductase (MDHAR), glucose-6-phosphate dehydrogenase (G6PDH), glutathione S-transferases (GST), glutathione peroxidases (GPX), glutathione reductase (GR), dehydroascorbate reductase (DHAR), ascorbate peroxidase (APX), polyphenol oxidase (PPO), and phospholipid hydroperoxide glutathione peroxidase (PHGPX) exhibit increased activity in plants [109]. Farooq and his team conducted a study that found stress tolerant cotton genotypes displayed increased enzyme activity of CAT, POD, and SOD [31]. This suggests that these enzymes may enhance stress tolerance. The study found that CAT and POD levels can serve as indicators of stress tolerance due to their role in breaking down harmful hydrogen peroxide byproducts [31].

## 6. Advanced Breeding in Cotton (Mutation Breeding)

A mutation is a heritable alteration in a living cell of DNA that is not generated via genetic recombination/segregation. The deliberate use of mutations in plant breeding is commonly known as "mutational breeding". Mutational breeding is employed to create the genetic variation in existing germplasm [1]. The mutation developed in an organism is determined by two main steps such as screening of the desired mutant followed by confirmation using various biological techniques. Firstly, mutagens such as chemicals, gamma rays, fast neutrons, as well as X-rays are bombarded onto seeds and then treated seeds are grown. The plants with desired characteristics are chosen and grown again for further segregation. To release a new variety by exploiting the mutational breeding, multiple trials are conducted at various locations [114].

Contrary to selection/hybridization, mutational breeding offers the privilege of correcting a defect in a crop that is otherwise excellent without sacrificing its agronomic or qualitative characteristics. Mutational breeding has found a position in plant breeding as a result of these benefits, dating back to the earliest release of mutant cultivars from fundamental mutation research in Europe. Chemical and physical mutation induction techniques have been improved in major crops, and mutant population selection methodologies have been developed. Newly discovered mutagens are being explored, such as cosmic rays, ion-beam radiation and a hitherto undiscovered spectrum of mutations have been uncovered; however, ionizing radiation and alkylating substances remain prevalent. 

The development of effective in vitro technologies for various crops, including cotton, has played a significant role in enhancing the efficiency of mutational breeding [115]. 

Crop evolution and plant breeding both rely heavily on various mechanisms such as genetic recombination, natural selection, and artificial selection. Polyploidy plays a crucial role in species evolution and formation by enhancing phenotypic diversity, heterosis, and resistance to mutations. Furthermore, allopolyploidization (interspecific hybridization) is considered more advantageous in evolution due to its remarkable heterosis effect, including increased biomass, growth rate, fertility, and stress resistance. As a result, tetraploid cultivated cotton species (*G. hirsutum* and *G. barbadense*) exhibit higher fiber quality and yield compared to cultivated diploid species (*G. arboreum* and *G. herbaceum*) [116]. Allopolyploidy, autopolyploidy, and aneuploidy are the three types of mutations involving chromosome numbers. For example, polyploids are expected to make up 50–70% of ornamental flower yields [117].

Allopolyploidy is the consequence of combining the genomes of two or more species; it is most often caused by intergeneric/interspecific crossing methods resulting from chromosome duplication. Many crops (including cotton) have followed this path. The existence of duplicated homologous allele pairs in allopolyploids complicates mutant selection; nevertheless, targeting induced local lesions in genome (*TILLING*) populations may help to detect and target these alleles for breeding purposes. Tomato, rice, sesame, maize, and barley are a few examples of crops that have evolved at the diploid level [115].

Furthermore, the mutation resulted in a rapid harvest due to a quick flowering burst. For instance, cotton’s early and consistent blooming may also be exploited to make automated picking easier. The discovery of flowering locus T (FT) became well-known because of its application in advanced breeding projects [118]. FT is a tiny globular protein that travels to sieve elements after interacting with FT-interacting protein 1. For nuclear localization, FT is transported from sieve elements toward the shoot apical meristem; here, it binds with phospholipid phosphatidylcholine and *bZIP* transcription factor FD which initiates the flower development by expressing and suppressing particular genes [119]. 

Studies support the notion that low-dose irradiation stimulates growth by altering the hormonal signaling network in plant cells or by boosting cells’ antioxidant capacity to overcome stress conditions [120]. Moreover, low doses of mutation produce genetic variation such as plant height, boll numbers, yield, ginning out turn %, seed index, harvest index, as well as fiber traits in cotton [121,122]. Rana Saeed and his colleagues developed the variety named NIAB-78 (http://www.niab.org.pk/, accessed on 5 October 2022) was successfully created using mutation breeding and has early maturity with high yield. Another successful example of mutation breeding is NIAB 92, created by using gamma rays emitted from ^60^CO [123]. Both NIAB-999 and NIAB-111 were resistant to heat stress and cotton leaf curl virus with high yield. These two mutants were also developed using mutational breeding. NIAB-78 was crossed with REBA-288 to produce NIAB-777. The pollen of REBA-288 was irradiated before crossing. In summary, mutational breeding has potential to create additional genetic variation in existing germplasm [124].

## 7. Molecular Breeding: Marker-Assisted Selection (MAS)

Genetic characterization of germplasm to identify genes that enhance agronomic traits can aid in the development of crops that are better suited to changing climate conditions. Investigating the genetic mechanisms of phenotypic variation under salt stress using molecular marker-based quantitative trait association [125] has the potential to improve the efficiency of breeding programs [126,127]. Currently, genetic map creation research mainly employs three major DNA-based molecular markers: simple sequence repeats (SSR) [128,129], single-nucleotide polymorphism (SNP) [130,131], and intron length polymorphisms (ILD) [132].

Chee and Campbell in 2009 found that molecular biology methods have gained significant attention in the last 15 years for exploring the structure, function, and evolutionary relationships of the cotton genome [93]. Developing the basic infrastructures of polymorphic DNA markers, unique genetic mapping populations, and comprehensive genetic linkage maps took 15 years. They also identified the location, importance, and intricacy of the QTL related to fiber properties. Researchers use traditional and molecular genetics to study fiber quality. After 15 years of molecular genetic research, scientists have a better understanding of the genes responsible for the genetic basis of cotton fiber quality. Most of the loci controlling fiber quality characteristics are concentrated in "gene islands" that are not randomly distributed throughout the A and D genomes. Classical breeding and molecular markers can work together to enhance the effectiveness of crop improvement programs. Classical breeding involves selecting plants with desirable traits, while molecular markers provide more precise tracking of the inheritance of these traits, resulting in better-informed breeding decisions [93]. Domesticated cotton has its roots in perennial wild cotton, which has adapted to semi-arid, subtropical environments marked by repeated drought and severe temperatures, making cotton an exceptional model for studying the molecular foundations of plants’ responses to water scarcity and salt stress.

Quantitative trait mapping, along with MAS and transgenic breeding, is practiced today. Molecular breeding is expected to yield gains in various areas, primarily due to a deeper understanding of plant genomic structure. Improving cotton cultivars is crucial, especially when they face high salt concentrations or water scarcity. For many cotton breeders, a crucial goal is to breed varieties that achieve a balance between yield and fiber quality under various conditions to meet the demands of their target markets. Techniques such as MAS have effectively enhanced the harvest index and yield ratio to whole plant weight under salt stress [133]. MAS and QTLs are useful molecular tools [133]. With the advent of MAS, cotton germplasm’s intra- and inter-variations can be employed to produce salt resistant varieties [34].

## 8. Different Markers

Under salt stress, distinct genes are expressed for various purposes such as cellular component, molecular function, and biological processing in cotton [134].

- DNA-based markers display Mendelian inheritance, unlike phenotypic markers, and have no environmental or epistatic effects. They are beneficial in tagging, cloning, introgression of useful genes from exotic genetic resources, and QTLs for specific trait enhancement. However, combining traditional breeding with modern molecular approaches will be highly useful in the development of salttolerant cultivars [26].
- Stress is a genetic condition that prevents full expression of genes. A wide variety of molecular markers are accessible for crop study. DNA-based markers are classified according to their application: RAPD markers are generated through the amplification of unknown DNA sequences using short, arbitrary oligonucleotide primers, making them a valuable tool in situations where information about the DNA sequence is not known beforehand in molecular biology [135,136]. On the other hand, simple sequence repeats (SSRs) are commonly employed for the purpose of characterizing plant varieties and analyzing their diversity, particularly in cultivated species where polymorphism levels are relatively low [2].
- These markers are also used in the QTL mapping to identify stress linked genes. Salt stress and drought reactions are regulated by basic genes called dehydrins and saltol [137]. Moreover, in inbred lines, single-gene SNPs markers allow sequencing of stress-related traits and genetic mapping. Advanced methods and marker modification allow for marker-assisted breeding to improve abiotic stress tolerance [137].
- DNA markers may be exploited to identify parents, analyse genetic diversity, and identify, confirm, and construct genetic linkage groups with high precision. Crop genetic analysis uses a wide variety of molecular markers. These markers are classified as either PCR-based or non-PCR-based indicators. DNA markers known as restriction fragment length polymorphisms (RFLPs) are produced through hybridization. In the 20th century, these markers were widely employed for gene mapping and other genetic studies in molecular biology. The PCR (polymerase chain reaction) method was invented by Cary Mullis in 1983. It enables the amplification of a small amount of DNA without the need for living organisms [138]. DNA marker system was influenced by their work and their usage in genomics. Utilizing PCR-based genetic markers decreased the time and cost of genetic mapping using probe hybridization [139]. PCR is an in vitro method for amplifying gene or locus DNA. The sequence of oligonucleotide primer should be complementary to gene present in the vicinity of that primer. From a little bit of a single design, melting and repetitive DNA replication creates a large quantity of interesting sequences. The information about sample gene sequence is required for PCR based markers, for instance, sequence-characterized amplified regions (SCAR), SSR, and SNPs [140].
- Salt tolerance in crops may be assessed using DNA-based molecular markers. Intron length polymorphism (ILP), RFLP, SSRs, expressed sequence tags and simple sequence repeats (EST-SSRs) all have shown to be expedient for swift and subtle screening [141]. However, advancements in high-throughput sequencing technology have made SNPs the preferred marker for salt tolerance investigations [142]. The discovery of chromosomal areas linked with salt stress resistance opens up new possibilities in MAS breeding and may be used for enhancing resistance towards salt stress [141].
- Sheidai and others in 2018highlighted the importance of agronomically fit, high yielding, drought- and salt-tolerant cotton varieties [143]. For hybridization purposes, the selection of appropriate parental genotypes, genetic and agronomic diversity within salt, and drought-tolerant cultivars must be studied. Some genotypes contain private bands that correspond with salt or drought tolerance. An analysis of molecular data revealed that certain genotypes shared genetic affinity, while others were genetically distinct. Malik and his colleagues in 2014revealed that various crossing combinations among the groups result in salt and drought tolerant cotton. An efficient, cost-effective, and rapid method of genetic fingerprinting for a large germplasm is using molecular markers such as inter retrotransposon-amplified polymorphism (IRAP), sequence-related amplified polymorphism (SRAP), and retrotransposon microsatellite amplification polymorphisms (REMAP) in cotton genotyping [144].

Lin and other fellows in 2005 used a combination of RAPDs, SRAPs, and SSRs to construct a genetic map of cultivated cotton and understand the genetic basis of cotton fiber characteristics to enhance fiber quality. In the study, 238 SRAP primer combinations, 368 SSR primer pairs, and 600 RAPD primers (107 RAPDs, 437 SRAPs, and 205 SSRs) were used to evaluate polymorphisms among *G. hirsutum* cv. Handan208 and *G. barbadense* cv. Pima90. The genotyping was performed on 69 offspring of the F_2_ generation resulting from an interspecific cross between “Pima90” and “Handan208”. Out of the total of 566 loci, 41 linkage groups were identified, each containing three or more loci. Additionally, 28 linkage groups were assigned to SSR markers with known chromosomal locations. It spanned 5141.8 cM with a 9.08 cM inter locus gap. A total of 135 loci (18.0 percent) showed asymmetrical segregation while most contained a surplus amount of maternal paternal alleles. There was a total of 13 QTLs identified associated with fiber characteristics: 2 for strength, 7 for micronaire value and 4 for length. The identified QTLs accounted for 16.18–28.92 percent of trait variance, with six QTLs found in the A subgenome, six in the D subgenome, and one unassigned linkage group. To enhance the micronaire value, a molecular marker-assisted selection method could use three QTLs located on LG1 [145].

In 2010, Zhang and others s utilized 88 SSR markers to analyze the genetic diversity of cotton germplasm that is linked to salinity. They detected a total of 338 alleles at the 88 SSR loci, with 312 alleles identified in salt susceptible germplasm and 333 alleles in salt tolerant germplasm. The mean values for polymorphism information content (PIC), average genotype diversity index (H′) and average effective number of alleles (Ne) in salt-tolerant germplasm were 0.613, 1.083, and 2.929, respectively. On the other hand, for susceptible germplasm, the mean values for Ne, PIC, and H′ were 2.883, 0.605, and 1.071, respectively. Both salt tolerant and salt sensitive germplasm had comparable similarity coefficients. The values were in range of 0.530 to 0.979 in salt tolerant varieties (from 0.525 to 0.878). Variety clustering revealed one main and two minor groupings. Chemometric analysis of Chinese salinity tolerant germplasm revealed limited pedigrees within the group. These findings can help to evaluate cotton pedigrees, enhance cotton hybrids, and eventually increase the adoption of salt resistant germplasm [146].

In 2012, Abdi and his colleagues used 14 ISSR loci to generate 65 polymorphic DNA fragments. The ISSR markers enabled the clustering of 28 cotton cultivars into three distinct groups. Regression analysis of the three salt treatments stress revealed that 23, 33, and 30 markers were associated with the evaluated characteristics. These markers could potentially aid breeders in enhancing the salt stress resistance of cotton cultivars through marker-assisted selection. [147]. The details of markers linked with salt stress are provided in Table 2.

## 9. Genotype by Sequencing (GBS) and Genome-Wide Association Studies (GWAS)

GBS is a method in which sequences are utilized concurrently to identify and score SNPs, consequently skipping the full step for the development of marker assay [162]. The use of next-generation sequencing (NGS) technology has resulted in significant advancements in whole-genome sequencing, providing the capacity for ultra-high-throughput sequencing. This breakthrough is expected to revolutionize plant breeding and genotyping. The GBS technique has been developed and applied to sequence multiplexed samples, integrating molecular marker identification with genotyping. This approach extends the potential of NGS in the study of large crop genomes such as wheat and maize [163].

Identifying the genetic basis of salt tolerance is an urgent priority for the development of salt tolerant varieties. Latyr Diouf and his team during 2017 generated a genetic map utilizing 5178 filtered GBS markers and 277 F_2:3_ populations. Their study involved an intraspecific cross between two upland cotton accessions, CCRI35 (salt tolerant) and Nan Dan Ba Di Da Hua (NH) (salt sensitive), which are commonly cultivated in China. The genetic map covered 4768.09 cM with an average distance of 0.92 cM, and 66 QTLs were identified for 10 salinity related traits across three salt treatments (0, 110, and 150 mM). Out of the 66 QTLs, only 14 were consistently detected, explaining 2.72% to 9.87% of the phenotypic variation. The salt sensitive parents contributed 10 QTLs, while the salt tolerant parents contributed 4 QTLs, with a 3:1 parental contribution ratio. Of the 14 consistent QTLs, five were located in the At sub-genome, and nine were in the Dt sub-genome. Additionally, the study identified 8 clusters containing 12 potential key genes related to salt stress [159].

In this study, 217 upland cotton cultivars were evaluated for salt tolerance related traits over a 2-year period using GBS in a GWAS. A total of 51,060 SNPs across 26 chromosomes were analyzed, resulting in the identification of 25 significant associations with three salt tolerance related traits. Chromosomes A13 and D08 displayed stable and expressed associations with relative plant height, while chromosome A07 was associated with relative shoot fresh matter weight and chromosomes A08 and A13 were associated with relative shoot dry matter weight. The integration of GWAS and transcriptome analysis identified 12 salt induced candidate genes, of which three were selected for functional verification. The results showed that silencing *GH_A13G0171* in plants increased salt tolerance traits, indicating a negative role in regulating the salt stress response [157].

Reduced representation genotyping is commonly utilized in agricultural genetics and breeding, and one such tool is genotyping-by-sequencing (GBS). This approach decreases genome complexity by utilizing barcoding restriction enzymes to sequence only a subset of DNA fragments on a high-throughput next-generation sequencing (NGS) instrument. Bioinformatics analysis utilizes sequence reads that are indexed to locate genetic variations, and a sample-by-variant matrix is used to evaluate genetic diversity. This makes it convenient and cost-effective to gauge genetic variation in populations. Since the first molecular marker-based genetic map of cotton was published in 1994, researchers have discovered many QTLs that govern essential agronomic characteristics such as fiber quality, yield, and disease resistance. However, only a limited number of studies have investigated salt tolerance in cotton [157].

GWAS have recently been employed as an effective tool for dissecting the genetic basis of various phenotypic traits in genetically heterozygous populations [164,165]. By employing statistical analysis, this approach assesses the association between genotype and phenotype, uncovering the alleles, candidate genes, and molecular markers that contribute to specific traits [166]. This technique is currently applied to multiple crops, including cotton (*Gossypium hirsutum* L.), maize (*Zea mays* L.), rice (*Oryza sativa* L.), and soybean (*Glycine max* L.), to identify the precise chromosomal locations and characterize numerous potential candidate genes that are accountable for salt stress. The use of GWAS in numerous crops has garnered significant attention due to challenges arising from the complexity of their genomes and inadequate genetic data [167]. Nonetheless, significant strides in cost-effective NGS and advancements in accurate resequencing using innovative GBS techniques have facilitated GWAS in diverse crops [168,169].

**Figure 1:**
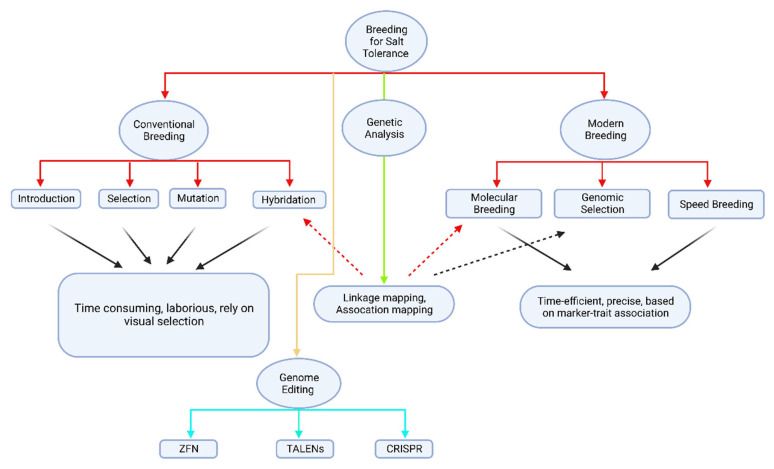
Schematic representation of combinations of various approaches for developing salt tolerant cotton.

Presently, there are multiple GBS techniques that can be broadly classified into two categories: methods based on genome complexity reduction, such as those using restriction enzymes and transcriptomes, and methods based on target enrichment or capture, such as PCR amplification, molecular inversion probes (MIPS), and hybrid capture [170]. However, target capture approaches are usually more prevalent when genomic resources are accessible since they can only select the sequence of interest in genomic region, typically with more accuracy and durability [171]. For some plant species with complex and extensive genomes, the cost per sample and the overall depth of coverage necessary to impact the genotyping process make whole-genome and exome sequencing impractical, despite their usefulness in detecting new genetic variations [172]. The sheer magnitude of data generated may also present novel challenges in terms of computer processing and management [173]. Consequently, many researchers studying plant, human, and animal species have acknowledged target capture sequencing as a feasible approach, selectively focusing on sequencing exons, specific variant regions, or genes of interest [174]. The assay is highly resilient and economical, and can deliver more comprehensive sequencing coverage as needed. As a result, GBS is now commonly employed in genomic selection (GS), GWAS, and various functional genomic investigations [167]. Conventional breeding has limitations, including being time-consuming, having limited genetic diversity, low efficiency, dependence on environmental conditions, and difficulty breeding for complex traits. Therefore, it is essential to integrate conventional breeding with other approaches to cultivate salt tolerant/resistant cotton varieties capable of withstanding the growing threat of salt stress caused by changing climate scenarios. Although conventional and modern breeding technologies for developing climate resilient crops have their limitations, their combination is essential to tackle the current challenges associated with the decline in cotton production. In this regard, we have provided a schematic flowchart (Figure 1) that shows how breeders can utilize both conventional as well as modern breeding technologies for developing smart and climate resilient cotton.

## 10. Future Prospective

### 10.1. CRISPR/Cas9

Clustered Regularly Interspaced Short Palindromic Repeats - CRISPR associated protein 9 (CRISPR/Cas9) is widely regarded as a highly effective tool for genome editing in numerous important crops, owing to its superior efficiency, cost-effectiveness, and ease of use when compared to other genome editing techniques such as Transcription Activator-Like Effector Nucleases (TALENs) and Zinc Finger Nucleases (ZFNs). CRISPR/Cas9 has revolutionized biological research, as it is site-specific and edits the genomic region of interest with higher precision in both simple and complex organisms. Although substantial work has been carried out to enhance its efficiency and target specificity, there is a need to carry out more research to improve its efficacy. Even though CRISPR/Cas9 technology has a number of limitations that restrict widespread use, many techniques are being investigated to enhance its efficacy for modifying human, plant, and animal cells [175].

### 10.2. Base Editing (BE) 

BE is a new and fast technique in genome editing that allows nucleotide replacements without a double strand break (DSB) or donor template. In the past three years, successful adenine deaminase-based base editors were developed and used in both animals and plants. Cas9 variations can be employed for upgrading the current cytosine base editor (CBE) and adenine base editor (ABE) base editors. Some base editors and cytidine deaminase mutants can improve DNA selectivity and reduce off-target activity. The highly precise base editors may improve the efficiency of precision breeding of crops. A lot of work has to be carried out to optimize, expand, and enhance the efficiency of developing base-editing technologies [176].

### 10.3. Prime Editing 

Until the arrival of prime editing, delivering a variety of editing applications with a single technology in a living system at high resolution was a critical task. With prime editing’s enormous potential in precise genome editing, we may expect substantial development in plant biological research in the coming years. To fully utilize primary editing in plant biology, several obstacles must be addressed, including the small size of the editing window, low efficiency, tissue, species specificity, and unknown cell [139].

### 10.4. Multiplex Editing

Multiplexed genome editing, made possible by CRISPR-based technologies, has the ability to revolutionize complex biological research and therapies. Combining CRISPR-guided genomic integration with bacterial and yeast genome engineering techniques may enable genome-scale engineering. These methods are essential for large-scale genome engineering initiatives such as GP -write (Genome Project Write) and de-extinction attempts. These future uses necessitate significant changes in editing, donor material creation, and distribution. Advancements in these aspects will usher in a new era of genomic biology when researchers can change genomes on a huge scale [147].

### 10.5. Noncoding RNA (ncRNAs)

The findings highlight the importance of using ncRNAs as biomarkers for diagnosis, prognosis, and treatment outcome prediction. As miRNAs have a large influence, a "molecular diagnostic database" might be beneficial. Novel deep sequencing technologies may aid in converting lab potential into clinical practice. ncRNA inhibition and reactivation will mark the conclusion of the discovery chain and result in a therapeutic approach. Synthetic miRNAs might be tailored to a patient’s genetic profile to control gene expression [177]. The expression of genes relevant to salt stress was regulated by the long noncoding RNA LncRNA973, which, in turn, affected cotton’s responses to salt stress. The findings pave the way for further research into lncRNA973-mediated responses to salt stress in cotton [178].

## Figures and Tables

**Table 1:** Impact of salt stress on different developmental stages of cotton.

Species	Variety	Experimental Condition	Stress Applied	Stress Duration	Salt Stress Effect on Cotton	References
*Gossypium hirsutum* L.	Xinluzao13 and Klebsiella oxytoca Rs-5	Pot	5 g NaCl	4 weeks	Reduced germination rate	[47]
*Gossypium barbadense* L.	Giza 90	Pot	Diluted sea water	10 days	Reduced growth	[48]
*Gossypium hirsutum* L.	GXM9	Petri dishes	150 Mm NaCl	6 days	Reduced germination	[49]
*Gossypium hirsutum* L.	9807 and Z010	Hydroponic culture	150 Mm NaCl	14 days	Reduction in growth	[50]
*Gossypium hirsutum* L.	Guoxin No.9	Hydroponic culture	150 Mm NaCl	12 days	Decreased plant height	[51]
*Gossypium hirsutum* L.	−	Pot	150 Mm NaCl	40 days	Growth Reduction	[52]
*Gossypium hirsutum* L.	BRS Topázio, BRS Safira, BRS Rubi	Pot	90 Mm NaCl	Vegetative and Flowering stage	Reduction in fiber quality and yield	[53]
*Gossypium hirsutum* L.	Nongfeng 133	Barrel Planting	7.3 g kg^−1^ salt stress	6 months	Reduced boll number and size	[49]
*Gossypium barbadense* L.	Giza 45	Mini-rhizotron System	150 Mm NaCl	Two Weeks	Reduced root length	[54]
*Gossypium hirsutum* L.	Nongfeng 133	Field	Brakish water irrigation	Throughout study	Reduced yield	[55]
*Gossypium hirsutum* L.	IR-NIBGE-13 and BS-2018	Pot	200 Mm NaCl	One week	Reduced plant biomass	[5]
*Gossypium hirsutum* L.	Xinluzao 52	Field	NaCl and CaCl_2_ (8.04 dS m^−1^)	30 days	Reduction in biomass	[56]
*Gossipyum barbadense* L.	−	Pot	Diluted sea water (EC = 52 dS m^−1^)	Throughout study	Decreased boll weight	[57]
*Gossypium hirsutum* L.	C-6524	Petri dishes	100 Mm NaCl and 100 Mm Mg_2_So_4_	5 days	Low germination	[58]
*Gossypium hirsutum* L.	Zhongmian 41	Hydroponic system	150 mM NaCl	20 days	Reduced plant growth	[59]
*Gossypium hirsutum* L.	CCRI 35, Z 51504 and CCRI 49	Pot	150 mM NaCl	9 days	Reduced shoot dry weight	[60]
*Gossypium hirsutum* L.	Simian 3	Pot	150 mM NaCl	30 days	Reduced plant biomass	[61]
*Gossypium hirsutum* L.	CCRI-79 and Simian 3	Field	11.46 dS m^−1^ Soil salinity	Throughout study	Reduced fiber quality	[38]
*Gossypium hirsutum* L.	Lu-mian-yan No. 24 and Xin-lu-zao No. 45	Pot	0.4% NaCl	Throughout study	Decreased biomass	[62]
*Gossypium hirsutum* L.	Zhong 07 and Zhong G5	Hydroponic system	200 mM NaCl	48 h	Stem binding	[62]
*Gossypium hirsutum* L.	Xinluzhong-37	Pot	150 mM NaCl	35 days	Reduced growth	[63]
*Gossypium hirsutum* L.	−	Pot	400 mM NaCl	12 h	Inhibited growth	[64]
*Gossypium hirsutum* L.	−	Field	Salt affected soil < 7.7 dS m^−1^.	Throughout study	Poor seedling emergence and fiber yellowness	[65]
*Gossypium hirsutum* L.	NIAB 777	Pot	NaCl (17 dS m^−1^)	40 days	Decreased plant height	[66]
*Gossypium hirsutum* L.	−	Paper germination test	250 mM NaCl	12 days	Poor germination rate, reduced seedling growth	[67]

**Table 2:** Identification of markers linked with salt tolerance in cotton.

Cotton Cultivar	Stress	Marker	Approach	Result	Salt Tolerant	Reference
*Gossypium tomentosum* with *Gossypium hirsutum*	150 mM NaCl	SSR	QTL mapping	11 QTLs, 5 candidate genes related with salinity	−	[148]
Upland cotton cultivars	200 mM NaCl	SNP	GWAS	Identification of 43 QTLs related with salinity	Acala Ultima, Deltapine Acala 90, M240 and Coker 315	[149]
Upland cotton cultivars	150 mM NaCl	SSR	Association mapping	Identification of 13 advanced salt tolerant cultivars	−	[128]
Upland cotton cultivars	200 mM NaCl	SSR	QTL mapping	Identification of 55 QTLs related to salt stress	−	[150]
Nongdamian 13 × Nongda 601	0.3% NaCl	SNPs	QTL mapping, resequencing and gene silencing	A stable QTL qSalt-A04-1 for salt tolerance, identification of two candidate genes, *GhGASA1* and *GhADC2*, related to salt tolerance	ND13	[151]
Xinza 1′ (GX1135 × GX100-2)	85 mM Saline ground water	SSR	QTL mapping	Identification of 3 QTLs related to salt stress, 7 genes related to related to salt stress	−	[152]
Upland cotton cultivars	0.3% saline solution	SNPs	GWAS	23 SNPs associated with salt tolerance; 6 putative genes associated with salt tolerance	Suwu 77-702	[131]
Upland cotton cultivars	100 and 200 mM NaCl	SSR	Association mapping	Identification of markers BNL3103 (D6), NAU478 (D8), and BNL3140 (D9) were associated with salt treatment	Jian mian 13, Si mian 4 and Gan mian 8	[153]
Asiatic cotton	150 mM NaCl	SNPs	GWAS	Identification of candidate genes (*Cotton_A_37775* and *Cotton_A_35901*) related to two key SNPs (Ca7_33607751 and Ca7_77004962) related to salt tolerance	GuangXiZuoXianZhongMian, LiaoYang-1, ZhaoXianHongJieMian, PingLeXiaoHua, KaiYuanTuMian, YuXi33, ChangShuXiaoBaiZi, PingGuoJiuPingZhongMian, FuChuanJiangTangZhongMian, TangShanBaiZiZhongMian, and ShiJiaZhuangJianMian	[154]
Upland cotton cultivars	0.4% NaCl	SSR	GWAS	Three salt stress associated SSR markers	−	[155]
Upland cotton cultivars	150 mM NaCl	SNPs	GWAS	2 SNPs (A10_95330133 and D10_61258588,2) associated with 20 putative genes related to salt tolerance	−	[156]
Upland cotton cultivars	350 mM NaCl	SNPs	GWAS	27 SNPs, 12 genes associated with salt tolerance	−	[157]
Upland cotton cultivars	150.0 mmol/L NaCl	SNPs	GWAS	27 SNPs associated with salt tolerance; six candidate genes related salt stress	−	[158]
Upland cotton lines	110, 150 mM NaCl	−	GBS-based QTL mapping	Identification of 14 stable QTLs and 12 putative gene associated with salt tolerance	−	[159]
Upland cotton cultivars	200 mmol/L NaCl	SNPs	GWAS-based approach	33 SNPs and 13 stable genes related to salt tolerance identified	−	[160]
Upland cotton cultivars	150 mM NaCl	−	QTL mapping	Identified 4 genes (*Gh_A04G1106, Gh_A05G3246, Gh_A05G3177*, and *Gh_A05G3266*)	−	[161]
